# Selection of the promising accessions of jamun (*Syzygium cumini* (L.) skeels) based on pomological characterizations

**DOI:** 10.1002/fsn3.3078

**Published:** 2022-09-23

**Authors:** Ali Khadivi, Farhad Mirheidari, Abdolvahid Saeidifar, Younes Moradi

**Affiliations:** ^1^ Department of Horticultural Sciences, Faculty of Agriculture and Natural Resources Arak University Arak Iran; ^2^ Ministry of Agriculture Jihad Sistan‐va‐Baluchestan Iran

**Keywords:** Breeding, Fruit, Genetic resource, Superiors, *Syzygium cumini*

## Abstract

Jamun (*Syzygium cumini* [L.] Skeels) is one of the most potential underutilized fruit crops. Here, phenotypic and pomological variability among 61 accessions of this species was investigated. Analysis of variance (*p* < .01) revealed significant differences among the accessions studied based on the traits recorded. Ripening date ranged from late June to mid‐July. Fruit color was purple in 13, dark purple in 30, and black in 18 accessions. Fruit weight ranged from 2.12 to 8.95 g, and fruit flesh thickness varied from 1.25 to 6.78 mm. Principal component analysis showed that fruit‐related characters are very important in differentiating among selections. The studied accessions were divided into two groups and several subgroups based on cluster analysis, which showed the phenotypic variations among them. Beside the significant differences among the accessions of different regions, significant variation was observed between the accessions of each region. The obtained results are useful for designing conservation strategies for the germplasm as well as implementing breeding programs, such as introducing cultivars with different goals, including early or late ripening and seedless, nonastringent, large, and deeper color‐fruits. Based on the fruit quality attributes, such as fruit weight, color, and taste; eight accessions, including Pirdan‐3, Soldan‐1, Pirdan‐6, Soldan‐5, Nasirabad‐3, Soldan‐3, Nasirabad‐8, and Ganjabad‐11, were selected which can be cultivated directly in orchards or used as parents in breeding programs.

## Graphical abstract

The obtained results are useful for designing conservation strategies for the germplasm as well as implementing breeding programs such as introducing cultivars with different goals such as early or late ripening and seedless, non‐astringent, large, and deeper color‐fruits. Based on fruit quality‐related traits such as fruit weight, color, and taste; eight accessions, including Pirdan‐3, Soldan‐1, Pirdan‐6, Soldan‐5, Nasirabad‐3, Soldan‐3, Nasirabad‐8, and Ganjabad‐11, were selected which can be cultivated directly in orchards or used as parents in breeding programs.

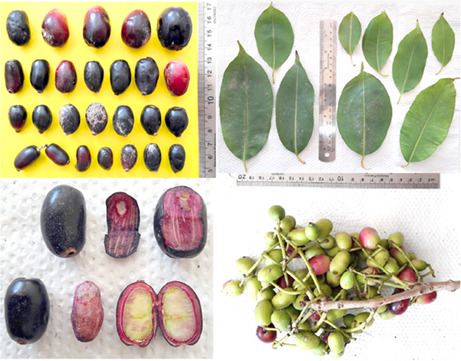

## 1 INTRODUCTION

*Syzygium cumini* (L.) Skeels, commonly called as “jamun” or “Indian black berry,” is one of the most potential underutilized fruit crops, native to the Indian subcontinent. Though the species has been found to grow throughout the tropical and subtropical regions (Singh et al., 2016), the same has been a neglected crop until the recent past. Over the past two decades, jamun has gained the consumer attention because of its exceptional health benefits. The drupaceous fruits of jamun are either consumed as fresh or processed into many products such as juice, squash, jam, etc. With the growing awareness on medicinal and nutritional properties, various jamun seed‐based formulations are gaining popularity among the common people (Singh et al., 2016).

Jamun fruits are a rich source of valuable compounds, such as anthocyanins, pectin, protein, and phenols. Their seeds contain the alkaloid jambosin and the glucoside jambolin or antimalin, which can reduce or eliminate the diastatic conversion of starch or sugars (Ayyanar & Subash‐Babu, 2012). Violet oil extracted from jamun seeds can be used as an effective agent against diabetes and heart and liver troubles (Sagrawat et al., 2006). The antioxidant activity of jamun fruits is due to their total phenolic compounds, which include anthocyanins. The main sugars in the ripe fruits of jamun are glucose and fructose with no trace of sucrose. Due to the fact that the medicinal properties of jamun fruits, especially their antidiabetic properties, have been determined, so in recent years, this fruit has been considered popular. The presence of malic acid, oxalic acid, gallic acid, and tannins has increased the medicinal value of the fruit. Also, the nutritional value of the jamun fruit is high. The fully ripe fruits with the taste of subacid spice flavor are eaten fresh and can also be used and processed into jam, jelly, squash, wine, vinegar, and pickle. Its foliage is used as feed for cattle. Its wood can be used in buildings, agricultural implements, and railway sleepers (Agarwala et al., 2019).

The demand for jamun fruit is increasing due to its nutritional and medicinal values. Therefore, the superior genotypes having high fruit quality and acceptable yield should be selected and introduced (Devi et al., 2019). Because jamun trees are propagated from seeds, there is considerable variation in the traits of the various organs of this plant. Also, being highly cross‐pollinated by nature, huge variability exists among the seedling populations of jamun. Variations are available in terms of fruit size, shape, pulp content, total soluble solids, and acidity which need documentation for identifying elite clones (Swamy et al., 2017). Characterizing the available germplasm to identify the elite genotypes of higher yield, better fruit quality, and adaptability is of utmost importance in jamun crop improvement programs. In addition to these fruit quality attributes, genotypes with dwarf tree stature, less vigorous types, and off season bearing need to be explored in view of area expansion and productivity in jamun (Khan, & Vaishali, Sharma, 2010). Thus, the present study was taken up to know the extent of variability existing among the accessions available in Sistan‐va‐Baluchestan province, Iran.

## 2 MATERIAL AND METHODS

### 2.1 Plant material

In the present study, phenotypic and pomological variability among 61 accessions of jamun (*S. cumini*) selected from six natural habitats in Sistan‐va‐Baluchestan province, Iran was investigated. The natural habitats studied included Soldan, Ganjabad, Rask, Nasirabad, Pirdan, and Shardar. Geographical coordinates and altitude corresponding to each surveyed area are presented in Table 1. To avoid the possibility of sampling and collecting clones of the selected accessions, the appropriate distance was considered between the accessions in each site.

**TABLE 1:** Geographical description for collection sites of *Syzygium cumini* accessions studied in Sistan‐va‐Baluchestan province, Iran

No.	Area	Latitude (N)	Longitude (E)	Altitude (m)	Sample size
1	Soldan	26°09′18″	61°47′05″	243	6
2	Ganjabad	26°25′45″	61°17′13″	595	15
3	Rask	26°13′51″	61°23′58″	389	11
4	Nasirabad	26°29′01″	61°12′56″	724	10
5	Pirdan	26°32′51″	61°13′06″	801	12
6	Shardar	26°35′17″	61°12′56″	841	7

### 2.2 The characters evaluated

Forty‐two morphological and pomological characters were used to determine phenotypic variability of the studied accessions (Table 2). A total of 50 leaves and 50 fruits per accession were randomly selected to evaluate the morphological and pomological traits. The traits, including leaf length, leaf width, petiole length, petiole width, fruit length, fruit width, fruit stalk length, fruit stalk diameter, seed length, and seed diameter, were measured using a digital caliper. Fruit and seed weight was measured using an electronic balance with 0.01 g precision. The remaining characters were qualitatively measured based on rating and coding (Table 3).

**TABLE 2:** Statistical descriptive parameters for morphological traits used to study *Syzygium cumini* accessions

No.	Trait	Unit	Min.	Max.	Mean	SD	CV (%)
1	Tree growth habit	Code	1	9	6.70	1.86	27.70
2	Tree growth vigor	Code	1	5	3.75	1.33	35.33
3	Tree height	Code	1	5	3.46	1.48	42.72
4	Branching	Code	1	5	3.46	1.23	35.64
5	Branch density	Code	1	5	3.56	1.37	38.57
6	Branch flexibility	Code	1	5	3.30	1.36	41.15
7	Trunk type	Code	1	7	2.80	1.92	68.64
8	Trunk diameter	Code	1	5	3.56	1.42	39.89
9	Trunk color	Code	1	5	3.26	1.57	48.16
10	Canopy density	Code	1	5	3.52	1.31	37.27
11	Tendency to form suckers	Code	1	5	2.28	1.32	57.81
12	Leaf density	Code	1	5	3.95	1.19	30.10
13	Leaf length	mm	117.45	178.93	150.74	18.98	12.59
14	Leaf width	mm	53.26	101.12	72.25	10.74	14.86
15	Petiole length	mm	12.21	28.98	21.08	3.36	15.93
16	Petiole width	mm	2.13	3.41	2.66	0.28	10.45
17	Leaf upper surface color	Code	1	3	2.25	0.98	43.42
18	Leaf lower surface color	Code	1	5	2.51	1.49	59.36
19	Vein color	Code	1	5	3.30	1.50	45.39
20	Leaf shape	Code	1	3	1.95	1.01	51.64
21	Leaf apex shape	Code	1	5	4.05	1.24	30.72
22	Leaf serration	Code	0	1	0.38	0.49	128.68
23	Leaf serration shape	Code	1	3	1.75	0.98	55.83
24	Leaf serration depth	Code	0	1	0.38	0.49	128.68
25	Ripening date	Date	Late June	Mid‐July	3.13	1.02	32.72
26	Fruit density	Code	1	5	3.33	1.47	44.11
27	Fruit shape	Code	1	5	2.44	1.27	52.13
28	Fruit length	mm	19.38	31.16	25.50	2.80	10.97
29	Fruit diameter	mm	12.57	23.36	17.34	3.00	17.31
30	Fruit stalk length	mm	1.17	4.75	2.73	0.77	28.40
31	Fruit stalk diameter	mm	1.15	3.60	2.32	0.50	21.63
32	Fruit weight	g	2.12	8.95	4.56	1.70	37.28
33	Fruit color	Code	1	5	3.16	1.43	45.19
34	Fruit flesh color	Code	1	5	2.70	1.31	48.44
35	Fruit flesh firmness	Code	1	5	3.23	1.47	45.36
36	Flesh thickness	mm	1.25	6.78	3.23	1.21	37.57
37	Fruit juice color	Code	1	5	3.39	1.50	44.19
38	Fruit taste	Code	1	5	2.74	1.29	47.08
39	Seed shape	Code	1	5	3.07	1.41	46.03
40	Seed length	mm	15.21	24.07	20.13	1.92	9.52
41	Seed diameter	mm	8.35	13.50	11.00	1.21	11.00
42	Seed weight	g	0.89	2.70	1.83	0.49	26.62

**TABLE 3:** Frequency distribution for the measured qualitative morphological characters in the studied *Syzygium cumini* accessions

	Frequency (no. of accessions)					
Trait	0	1	3	5	7	9
Tree growth habit	–	Weeping (1)	Spreading (3)	Open (16)	Semierect (25)	Erect (16)
Tree growth vigor	–	Low (6)	Moderate (26)	High (29)	–	–
Tree height	–	Low (11)	Moderate (25)	High (25)	–	–
Branching	–	Low (6)	Moderate (35)	High (20)	–	–
Branch density	–	Low (8)	Moderate (28)	High (25)	–	–
Branch flexibility	–	Low (10)	Moderate (32)	High (19)	–	–
Trunk type	–	Single trunk (25)	Multitrunk/Low (23)	Multitrunk/Moderate (7)	Multitrunk/High (7)	–
Trunk diameter	–	Low (9)	Moderate (26)	High (26)	–	–
Trunk color	–	Dark brown (15)	Gray (23)	Dark gray (23)	–	–
Canopy density	–	Low (7)	Moderate (31)	High (23)	–	–
Tendency to form suckers	–	Low (28)	Moderate (27)	High (6)	–	–
Leaf density	–	Low (3)	Moderate (26)	High (32)	–	–
Leaf upper surface color	–	Green (23)	Dark green (38)	–	–	–
Leaf lower surface color	–	Light green (26)	Green (24)	Dark green (11)	–	–
Vein color	–	Green‐yellow (13)	Light green (26)	Green (22)	–	–
Leaf shape	–	Oblong (32)	Elliptical (29)	–	–	–
Leaf apex shape	–	Mucronate (4)	Cuspidate (21)	Acuminate (36)	–	–
Leaf serration	Absent (38)	Present (23)	–	–	–	–
Leaf serration shape	–	Entire (38)	Undulate (23)	–	–	–
Leaf serration depth	None (38)	Low (23)	–	–	–	–
Ripening date	–	Late June (6)	Early July (45)	Mid‐July (10)	–	–
Fruit density	–	Low (12)	Moderate (27)	High (22)	–	–
Fruit shape	–	Ovate (23)	Oblong (32)	Elongate (6)	–	–
Fruit color	–	Purple (13)	Dark purple (30)	Black (18)	–	–
Fruit flesh color	–	Light purple (18)	Purple (34)	Dark purple (9)	–	–
Fruit flesh firmness	–	Low (13)	Moderate (28)	High (20)	–	–
Fruit juice color	–	Light pink (12)	Light purple (25)	Purple (24)	–	–
Fruit taste	–	Astringent (17)	Astringent‐sweet (35)	Sweet (9)	–	–
Seed shape	–	Ovate (14)	Oblong (31)	Elongate (16)	–	–

### 2.3 Statistical analysis

Analysis of variance (ANOVA) was performed to evaluate the variation among accessions based on the traits measured using SAS software (SAS® Procedures, 1990). Simple correlations between traits were determined using Pearson correlation coefficients (SPSS Inc., Chicago, IL, USA, Norusis, 1998). Principal component analysis (PCA) was used to investigate the relationship between accessions and determine the main traits effective in genotype segregation using SPSS software. The PCA is the simplest of the true eigenvector‐based multivariate analyses. Often, its operation can be thought of as revealing the internal structure of the data in a way that best explains the variance in the data (Iezzoni & Pritts, 1991). Hierarchical cluster analysis (HCA) was performed using Ward's method and Euclidean coefficient using PAST (Paleontological Statistics) software (Hammer et al., 2001). The first and second principal components (PC1/PC2) were used to create a scatter plot with PAST software.

## 3 RESULTS AND DISCUSSION

The ANOVA (*p* < .01) revealed significant differences among the accessions studied based on the traits recorded. Leaf serration and leaf serration depth showed the highest coefficient of variation (CV) (128.68% for both characters). Also, the CV was higher than 50.00% for trunk type, leaf lower surface color, the tendency to form suckers, leaf serration shape, fruit shape, and leaf shape (68.64%, 59.36%, 57.81%, 55.83%, 52.13%, and 51.64%, respectively). In contrast, seed length showed the lowest CV (9.52%), followed by petiole width (10.45%), fruit length (10.97%), seed diameter (11.00%), leaf length (12.59%), leaf width (14.86%), and petiole length (15.93%) (Table 2). Din et al. (2020) observed that the CV ranged from 14.36 (in seed length) to 46.16% (fruit flesh color) in a *S. cumini* germplasm from Pakistan.

The accessions were clustered into five groups based on the tree growth habit, including weeping (one accession), spreading (3), open (16), semierect (25), and erect (16). Branching, branch density, branch flexibility, and canopy density were predominantly moderate. Trunk was single in 25 accessions, while multi‐trunks were observed in the rest of the accessions, ranging from low to high. Tendency to form suckers was low (28 accessions), moderate (27), and high (6). Leaf shape was oblong (32 accessions) and elliptical (29), while leaf apex shape was mucronate (4), cuspidate (21), and acuminate (36) (Table 3). Leaf serration was absent in 38 and present in 23 accessions, and in those with having this trait, serration shape and serration depth were undulate and low, respectively. The range of leaf length and width was 117.45–178.93 mm and 53.26–101.12 mm, respectively. Petiole length varied from 12.21 to 28.98 mm, and petiole width ranged from 2.13 to 3.41 mm (Table 2). Anushma and Sane (2018) reported the range of 116.30–155.30 mm for leaf length, 50.40–75.30 mm for leaf width, and 15.20–26.30 mm for petiole length in a *S. cumini* germplasm from India.

Ripening date was late June in 6 accessions, early July in 45 accessions, and mid‐July in 10 accessions. Fruit density was low (12 accessions), moderate (27), and high (22). Fruit shape was ovate (23 accessions), oblong (32), and elongate (6) (Table 3). Din et al. (2020) observed ellipsoid, oblong, round, and oval shapes for fruit in a *S. cumini* germplasm from Pakistan.

Fruit color was purple in 13, dark purple in 30, and black in 18 accessions. Fruit flesh color was light purple (18 accessions), purple (34), and dark purple (9), while fruit juice color was light pink (12 accessions), light purple (25), and purple (24) (Table 3). Din et al. (2020) observed that fruit flesh color ranged from purple pink to pinkish white in a *S. cumini* germplasm from Pakistan.

Fruit taste was astringent–sweet in the majority of accessions (35), while astringent (17 accessions) and sweet (9 accessions) tastes were also observed. The range of fruit‐related characters was as follows: fruit length: 19.38–31.16 mm, fruit diameter: 12.57–23.36 mm, fruit weight: 2.12–8.95 g, and flesh thickness: 1.25–6.78 mm (Table 2). Anushma and Sane (2018) reported the range of 22.50–38.70 mm for fruit length, 16.30–34.20 mm for fruit diameter, and 5.58–11.18 g for fruit weight in a *S. cumini* germplasm from India. Besides, Devi et al. (2019) recorded the range of 17.11–43.66 mm for fruit length, 11.91–39.56 mm for fruit diameter, and 1.89–16.57 g for fruit weight in a *S. cumini* germplasm from India. Din et al. (2020) observed that fruit length, diameter, and weight varied from 22.00 to 41.70 mm, 14.00 to 29.70 mm, and 2.67 to 12.63 g, respectively, in a *S. cumini* germplasm from Pakistan.

**FIGURE 1:**
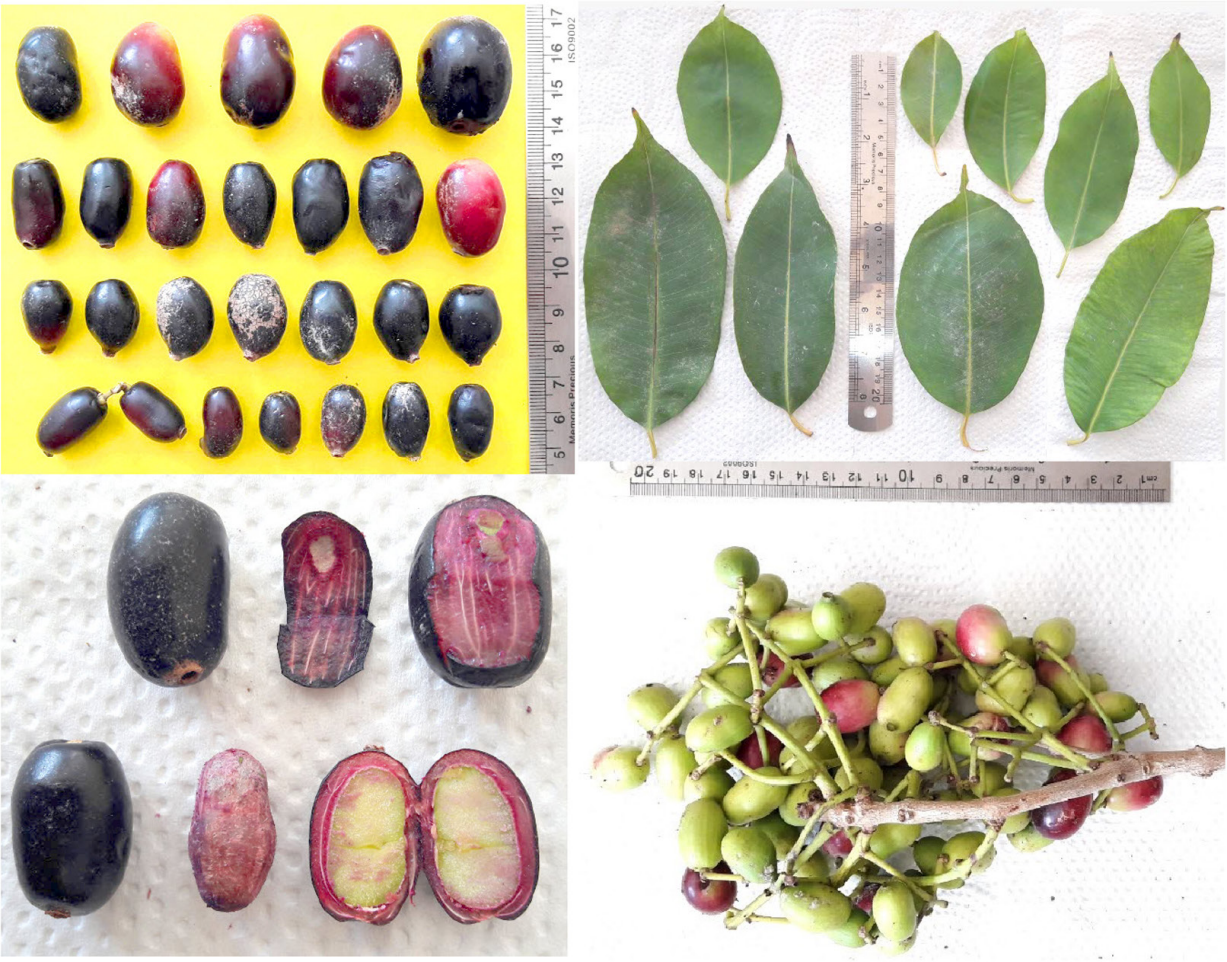
The pictures of leaves and fruits of *Syzygium cumini* accessions studied

Three types of seed shape were observed, including ovate (14), oblong (31), and elongate (16). The range of seed‐related characters was as follows: seed length: 15.21–24.07 mm, seed diameter: 8.35–13.50 mm, and seed weight: 0.89–2.70 g (Table 2). Anushma and Sane (2018) reported the range of 13.10–31.00 mm for seed length and 1.26–2.58 g for seed weight in *S. cumini*. Besides, Devi et al. (2019) recorded the range of 10.00–38.32 mm for seed length, 8.28–29.39 for seed diameter, and 0.74–3.90 g for seed weight in *S. cumini*. Din et al. (2020) observed that seed length, diameter, and weight varied from 14.30 to 26.00 mm, 5.70 mm to 18.00 mm, and 0.78–3.31 g, respectively, in a *S. cumini* germplasm from Pakistan. The pictures of leaves and fruits of *S. cumini* accessions studied are shown in Figure 1.

The positive and negative correlations were observed between the recorded characteristics (Table 4). Leaf length was positively correlated with leaf width (*r* = .79). Fruit weight was positively correlated with petiole length (*r* = .47), fruit length (*r* = .87), fruit diameter (*r* = .92), flesh thickness (*r* = .90), and seed weight (*r* = .78). Din et al. (2020) reported high correlations between fruit and seed dimensions and weights in *S. cumini*. When the measurement of a trait is expensive, complicated, time consuming, and difficult, other traits that have significant and high correlations with that desired trait can be indirectly used to measure it (Forde, 1975).

**TABLE 4:** Simple correlations among the quantitative morphological variables utilized in the studied *Syzygium cumini* accessions

Trait	Leaf length	Leaf width	Petiole length	Petiole width	Fruit length	Fruit diameter	Fruit stalk length	Fruit stalk diameter	Fruit weight	Flesh thickness	Seed length	Seed diameter	Seed weight
Leaf length	1												
Leaf width	0.79**	1											
Petiole length	−0.53**	−0.46**	1										
Petiole width	0.17	0.17	−0.16	1									
Fruit length	−0.49**	−0.34**	0.42**	−0.26*	1								
Fruit diameter	−0.39**	−0.31*	0.45**	−0.02	0.85**	1							
Fruit stalk length	0.04	−0.13	0.06	−0.01	0.24	0.25*	1						
Fruit stalk diameter	−0.13	−0.17	−0.16	0.04	0.22	0.04	0.06	1					
Fruit weight	−0.52**	−0.37**	0.47**	0.00	0.87**	0.92**	0.22	0.18	1				
Flesh thickness	−0.47**	−0.37**	0.51**	0.09	0.76**	0.91**	0.32*	0.05	0.90**	1			
Seed length	−0.19	−0.13	0.27*	−0.20	0.86**	0.74**	0.24	0.23	0.69**	0.55**	1		
Seed diameter	−0.03	−0.06	0.06	−0.04	0.56**	0.68**	0.11	0.02	0.55**	0.38**	0.72**	1	
Seed weight	−0.22	−0.10	0.25*	−0.05	0.76**	0.81**	0.02	0.18	0.78**	0.60**	0.84**	0.84**	1

* Correlation is significant at the .05 level

** Correlation is significant at the .01 level.

For the PCA, components with eigenvalues of more than 1.00 were retained to uphold reliability of the final output. Thus, 13 principal components (PCs) were observed which contributed 84.69% of total variance (Table 5). The values above 0.56 were considered to be significant for the studied traits. The PC1 explained 15.40% of total variance and was represented by fruit length (0.93), fruit diameter (0.93), fruit weight (0.90), flesh thickness (0.81), seed length (0.87), seed diameter (0.73), and seed weight (0.87) with positive correlations. Din et al. (2020) reported that fruit weight, fruit diameter, seed length, and seed weight were positively correlated with PC1 in a *S. cumini* germplasm from Pakistan, and suggested that these descriptors are very important in differentiating among selections. Fruit quality and yield‐related characters (fruit to pulp ratio, fruit size, etc.) have high economic concern (Andres‐Agustin et al., 2006) and can be used as target characters by plant breeders and growers (Mehmood et al., 2014). The PC2 explained 9.64% of total variance and was constituted by leaf serration (0.96), leaf serration shape (0.96), and leaf serration depth (0.96) with positive correlations. The third principal component (PC3) explained 6.93% of total variance and was represented by branch flexibility (0.64), leaf length (0.77), leaf width (0.70), and petiole length (−0.60). These characters were the most effective traits for separating and identifying the studied accessions. In addition, the fruit‐related traits are economically important and can also be applied as a useful tool for selecting accessions or new cultivars with superior traits. It has been reported that fruit‐related characters were important factors in differentiating and analyzing breeding materials dealing with the morphological characterization of *S. cumini* (Anushma & Sane, 2018; Devi et al., 2016; Devi et al., 2019; Din et al., 2020; Ningot et al., 2017; Singh & Kaur, 2016; Swamy et al., 2017).

**TABLE 5:** Eigenvalues of the principal component axes from the principal component analysis (PCA) of the morphological characters in the studied *Syzygium cumini* accessions

Trait	Component												
	1	2	3	4	5	6	7	8	9	10	11	12	13
Tree growth habit	−0.08	−0.11	0.09	0.14	0.83**	0.01	−0.14	−0.06	0.03	0.08	0.12	0.04	−0.11
Tree growth vigor	−0.07	−0.04	−0.08	−0.15	0.32	0.27	0.32	0.09	0.24	0.08	−0.24	0.35	0.26
Tree height	−0.03	0.14	−0.10	−0.19	0.73**	−0.18	0.32	0.06	0.13	0.06	0.03	−0.21	−0.04
Branching	−0.14	−0.10	0.04	−0.05	0.13	−0.06	0.13	−0.02	0.04	0.04	0.05	−0.87**	0.05
Branch density	−0.13	−0.07	−0.16	0.00	0.35	0.33	0.39	−0.15	0.24	−0.20	0.18	−0.44	−0.12
Branch flexibility	0.16	−0.13	0.64**	0.17	−0.51	0.09	0.13	0.15	−0.07	0.20	−0.06	−0.14	−0.01
Trunk type	0.13	0.11	0.11	0.20	−0.16	0.25	0.19	0.11	0.03	−0.20	0.12	−0.02	0.76**
Trunk diameter	−0.05	0.12	0.02	−0.18	0.35	0.21	0.14	−0.16	0.56**	−0.04	0.29	−0.17	−0.15
Trunk color	−0.01	0.39	−0.07	−0.15	0.67**	0.00	0.07	0.17	0.09	0.19	−0.15	−0.12	0.03
Canopy density	−0.09	0.23	0.18	−0.15	−0.09	0.15	0.75**	0.06	0.08	−0.21	0.19	−0.21	0.05
Tendency to form suckers	0.09	0.05	0.22	0.22	−0.12	0.16	0.27	0.73**	−0.10	−0.14	−0.03	0.16	−0.03
Leaf density	0.21	−0.17	−0.22	0.16	0.13	0.07	0.74**	0.03	0.11	0.09	−0.06	−0.02	0.08
Leaf length	−0.39	0.24	0.77**	−0.19	−0.03	0.11	−0.17	0.07	0.09	0.06	0.08	−0.02	0.02
Leaf width	−0.28	0.40	0.70**	−0.16	0.12	0.05	−0.09	0.14	−0.13	−0.14	0.12	0.15	0.06
Petiole length	0.37	−0.22	−0.60**	−0.08	−0.11	0.06	−0.30	0.24	0.08	−0.22	−0.04	0.03	0.14
Petiole width	−0.09	0.16	0.12	0.19	0.01	0.37	0.09	0.10	0.12	−0.10	0.11	0.00	−0.73**
Leaf upper surface color	0.09	−0.23	0.05	−0.03	0.06	0.06	0.04	0.15	0.03	−0.06	0.88**	−0.10	0.03
Leaf lower surface color	0.05	−0.23	−0.51	−0.28	0.25	0.09	0.24	0.14	−0.10	−0.02	0.35	0.36	0.04
Vein color	0.21	0.03	−0.21	0.01	−0.02	−0.20	0.15	−0.37	0.74**	0.03	0.17	0.12	0.00
Leaf shape	0.06	−0.04	−0.01	0.26	−0.03	0.03	−0.12	−0.62**	0.11	0.20	−0.54	−0.03	0.10
Leaf apex shape	−0.11	−0.04	0.05	0.09	0.18	0.11	−0.06	0.05	0.04	0.79**	−0.14	0.10	−0.01
Leaf serration	0.12	0.96**	0.12	−0.02	0.05	0.11	0.00	0.03	−0.01	0.03	−0.07	0.03	−0.01
Leaf serration shape	0.12	0.96**	0.12	−0.02	0.05	0.11	0.00	0.03	−0.01	0.03	−0.07	0.03	−0.01
Leaf serration depth	0.12	0.96**	0.12	−0.02	0.05	0.11	0.00	0.03	−0.01	0.03	−0.07	0.03	−0.01
Ripening date	0.07	0.31	−0.24	0.06	−0.02	0.18	−0.42	0.06	0.38	0.31	−0.04	0.33	0.37
Fruit density	0.28	−0.18	0.17	−0.64**	0.12	−0.09	0.18	−0.09	0.22	−0.22	0.30	0.14	−0.02
Fruit shape	−0.22	−0.16	−0.02	0.31	0.14	−0.79**	−0.08	−0.01	0.06	0.08	−0.02	−0.09	−0.06
Fruit length	0.93**	−0.02	−0.15	−0.02	0.01	−0.15	0.07	−0.06	0.08	−0.06	0.02	0.07	0.13
Fruit diameter	0.93**	0.10	−0.15	−0.04	−0.01	0.18	−0.02	0.09	0.12	−0.08	−0.02	−0.04	0.04
Fruit stalk length	0.21	−0.14	0.12	−0.20	0.18	−0.03	0.04	0.07	0.78**	0.09	−0.32	−0.12	−0.02
Fruit stalk diameter	0.18	−0.07	0.13	0.12	−0.18	−0.15	0.26	−0.74**	0.13	−0.18	−0.17	0.08	−0.15
Fruit weight	0.90**	0.04	−0.23	−0.03	−0.05	0.10	0.15	0.04	0.07	−0.15	−0.07	−0.02	−0.06
Fruit color	−0.44	0.10	−0.35	0.62**	−0.16	0.12	0.04	−0.16	0.08	0.22	0.11	0.12	0.12
Fruit flesh color	−0.04	−0.24	0.15	0.80**	−0.01	−0.39	0.02	−0.01	−0.06	−0.10	0.06	0.05	0.00
Fruit flesh firmness	0.02	0.35	−0.06	−0.40	0.03	0.01	0.39	0.41	0.14	0.25	0.13	0.17	−0.23
Flesh thickness	0.81**	0.09	−0.27	−0.01	0.00	0.12	0.06	0.19	0.22	−0.26	−0.13	−0.13	−0.04
Fruit juice color	−0.41	−0.03	−0.02	0.64**	−0.04	−0.22	0.18	−0.04	−0.26	−0.26	−0.19	0.12	−0.15
Fruit taste	−0.07	−0.31	−0.11	0.23	0.00	0.09	−0.03	0.19	−0.07	−0.60**	−0.02	0.34	0.08
Seed shape	−0.15	−0.17	−0.07	0.00	−0.02	−0.84**	−0.09	−0.22	0.02	−0.17	−0.04	−0.01	0.07
Seed length	0.87**	0.05	0.09	−0.15	−0.06	−0.08	−0.08	−0.17	0.08	0.13	0.10	0.13	0.10
Seed diameter	0.73**	0.09	0.17	−0.16	0.02	0.29	−0.06	−0.11	−0.07	0.37	0.16	0.14	0.04
Seed weight	0.87**	0.18	0.02	−0.18	−0.18	0.15	−0.01	−0.11	−0.13	0.12	0.09	0.16	0.02
Total	6.47	4.05	2.91	2.85	2.67	2.44	2.41	2.28	2.13	2.04	1.92	1.84	1.58
% of variance	15.40	9.64	6.93	6.78	6.35	5.80	5.73	5.43	5.08	4.85	4.58	4.38	3.76
Cumulative %	15.40	25.04	31.97	38.75	45.10	50.90	56.63	62.05	67.13	71.98	76.55	80.93	84.69

** Eigenvalues ≥0.56 are significant at the *p* ≤ .01 level.

**FIGURE 2:**
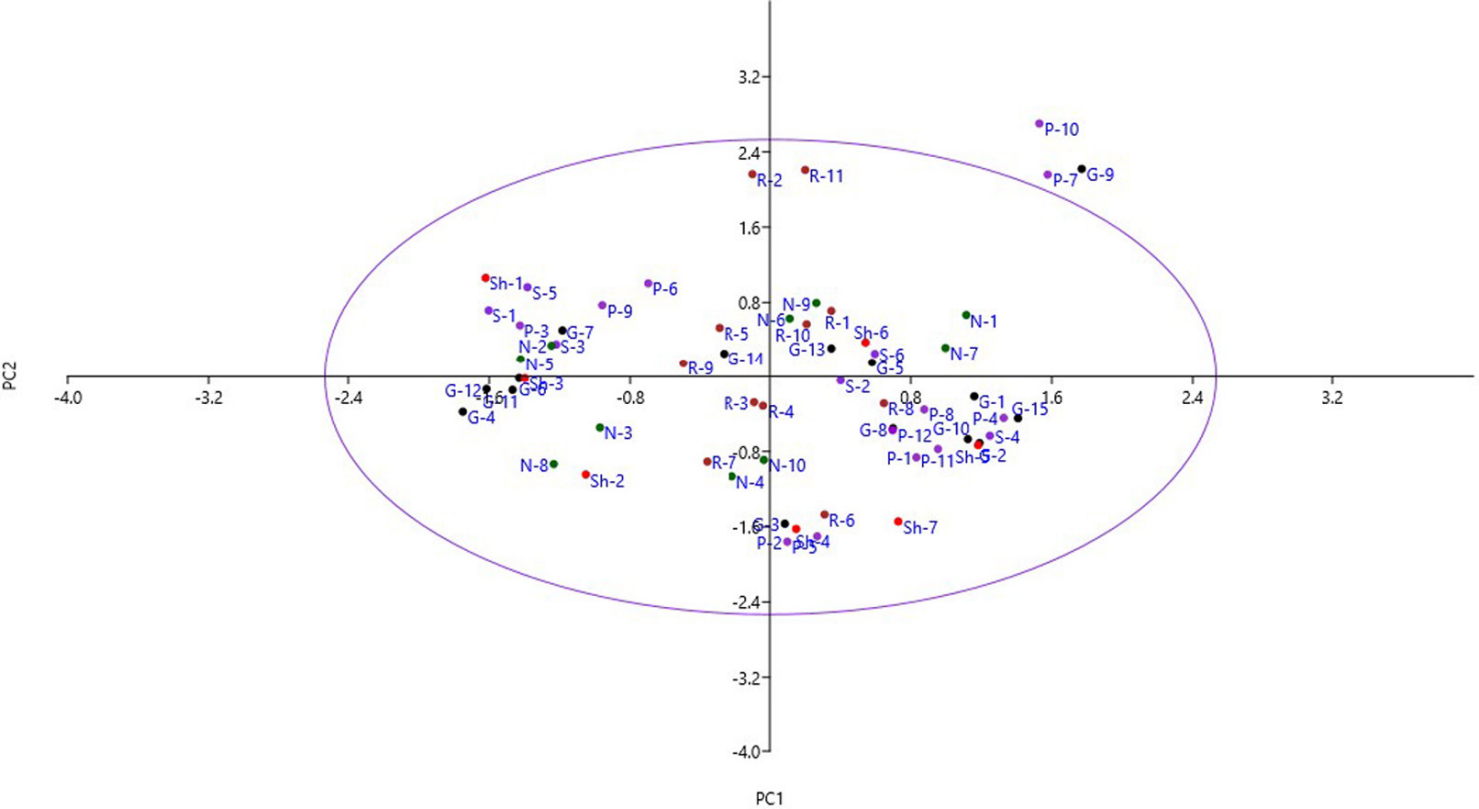
Scatter plot for the studied *Syzygium cumini* accessions based on first principal component/second principal component (PC1/PC2). The symbols represent the accessions of each area in the plot, including Soldan (S), Ganjabad (G), Rask (R), Nasirabad (N), Pirdan (P), and Shardar (Sh)

A dispersion biplot prepared according to PC1 and PC2 reflected the relationship among the accessions in terms of phenotypic similarity. The accessions were distributed into four sides of the plot and showed significant variations (Figure 2). By starting from negative toward positive values of PC1, the accessions showed gradual increases in fruit length, fruit diameter, fruit weight, flesh thickness, seed length, seed diameter, and seed weight. Furthermore, by starting from negative to positive values of PC2, the accessions indicated gradual increases in leaf serration, leaf serration shape, and leaf serration depth.

**FIGURE 3:**
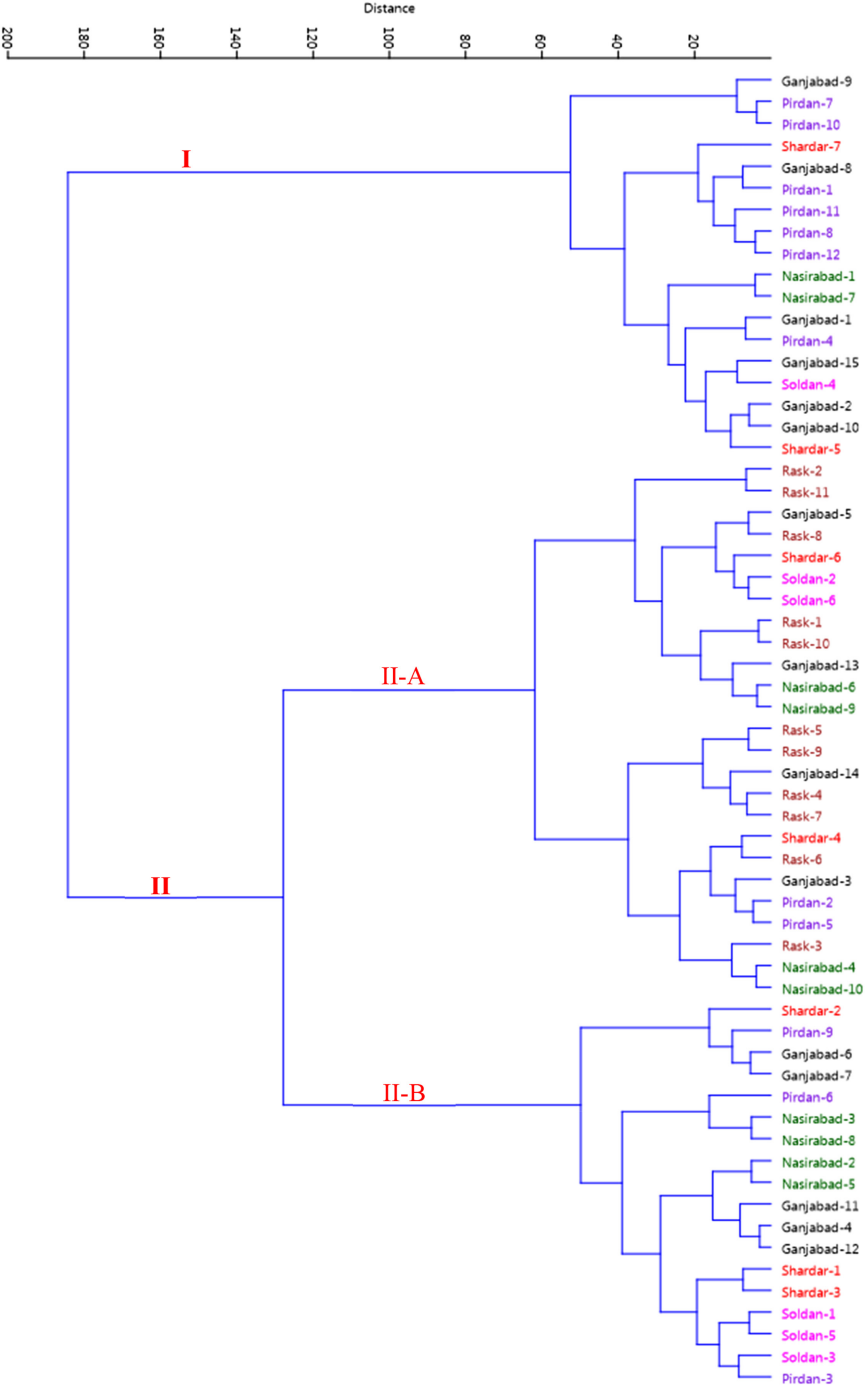
Ward cluster analysis of the studied *Syzygium cumini* accessions based on morphological traits using Euclidean distances

Cluster analysis based on the Ward's method showed two different major clusters among all the accessions studied (Figure 3). The first cluster (I) contained 18 accessions. The second cluster (II) consisted of the majority of the accessions studied and was divided into two subclusters with high diversity. Subcluster II‐A consisted of 25 accessions, while subcluster II‐B contained 18 accessions.

**FIGURE 4:**
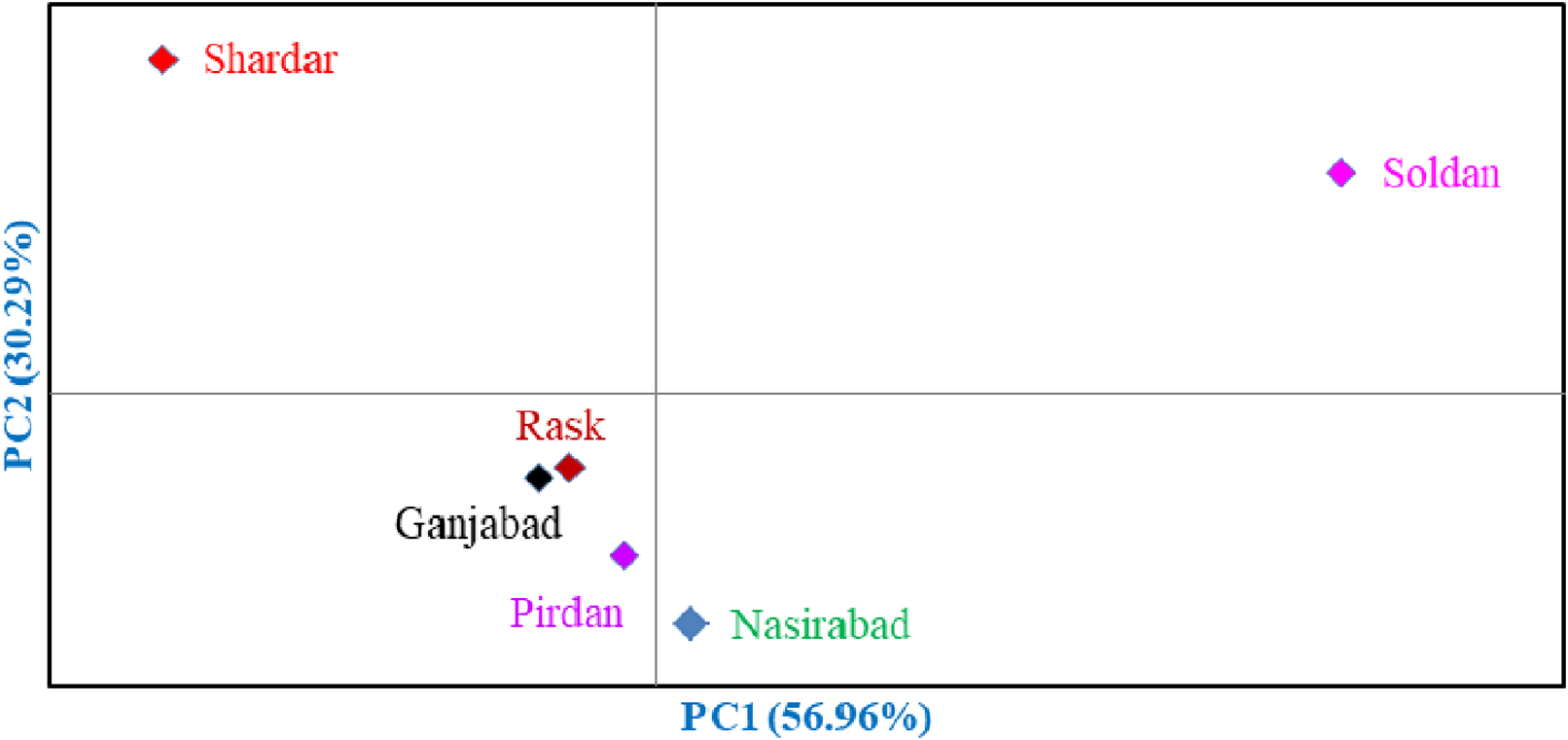
Biplot for the studied populations of *Syzygium cumini* based on the morphological characters

Besides, the association between the natural habitats studied was visualized in greater detail according to the morphological characters. Based on the biplot created using PCA of the population analysis, the studied six natural habitats formed four groups (Figure 4). Soldan area was placed in the first group, and Shardar area was placed in the second group. Also, Nasirabad formed the third group, while the fourth group consisted of rest of the areas, including Ganjabad, Rask, and Pirdan.

Broad phenotypic diversity existed among the studied *S. cumini* accessions. Beside significant variations among the accessions of different collection areas, great variability was found within the same varietal group. The considerable phenotypic variation observed here indicated that such germplasm is valuable genetic resource for *S. cumini* improvement. Therefore, it has become imperative to establish strategies for preserving wild *S. cumini* germplasm and conserving these genetic resources. Variation evident in case of qualitative and quantitative characteristics among accessions collected from same geographical area might be due to different genetic makeup (Karadeniz & Ekşi, 2002; Singh et al., 2015). These characters can be used as a tool for cultivar identification (Sharif et al., 2019). Fruit color and shape are best indicators of ripening and have been extensively used for cultivar identification in researches (Agrawal et al., 2017). Most dependent traits for fruit characterization are fruit apex, base, and shape (Sharif et al., 2019). Diversity in such traits was also observed as an important character for selection of plants by Bal et al. (1992) and Rodrigues et al. (2008). Several studies from other countries have been reported on morphological characterization of *S. cumini* collections (Devi et al., 2016; Ningot et al., 2017; Singh & Kaur, 2016; Swamy et al., 2017). While identifying the elite ones with better yield and fruit quality, emphasis needs to be given for dwarf types with compact canopy for effective utilization of limited land available (Anushma & Sane, 2018). Modern trend of selection and breeding for important cultivars has narrowed the diversity of several fruits (Mehmood et al., 2014; Sharif et al., 2019), and there is a prerequisite for exploration and evaluation of new sources of natural diversity in jamun with specific implication for improvement and germplasm management.

## 4 CONCLUSION

In the perennial fruit crops like jamun (*S. cumini*), elite clonal selection is mostly adopted for conventional crop improvement. The present data may help in the development of strategies for *S. cumini* germplasm management and may allow for more efficient use of this germplasm in future breeding programs. The findings of the present study are important for the management of germplasm resources and breeding studies, such as the development of cultivars with late or early ripening, nonastringent fruits, seedless fruits, large fruits, and fruits with deeper color. Based on the fruit quality attributes, such as fruit weight, fruit color, fruit flesh color, and fruit taste; eight accessions, including Pirdan‐3, Soldan‐1, Pirdan‐6, Soldan‐5, Nasirabad‐3, Soldan‐3, Nasirabad‐8, and Ganjabad‐11, could be selected for direct cultivation in the orchards and used in breeding programs.

## ACKNOWLEDGEMENT

None.

## CONFLICT OF INTEREST

The authors declare no conflict of interest.

## Data Availability

The data that support the findings of this study are available from the corresponding author upon reasonable request.
